# OGG1-DNA interactions facilitate NF-κB binding to DNA targets

**DOI:** 10.1038/srep43297

**Published:** 2017-03-07

**Authors:** Lang Pan, Wenjing Hao, Xu Zheng, Xianlu Zeng, Adeel Ahmed Abbasi, Istvan Boldogh, Xueqing Ba

**Affiliations:** 1 The Key Laboratory of Molecular Epigenetics of the Ministry of Education, Northeast Normal University, Changchun, Jilin 130024, China; 2 Institute of Genetics and Cytology, Northeast Normal University, Changchun, Jilin 130024, China; 3 Department of Microbiology and Immunology, Sealy Center for Molecular Medicine, University of Texas Medical Branch at Galveston, Galveston, Texas 77555, USA

## Abstract

DNA repair protein counteracting oxidative promoter lesions may modulate gene expression. Oxidative DNA bases modified by reactive oxygen species (ROS), primarily as 7, 8-dihydro-8-oxo-2′-deoxyguanosine (8-oxoG), which is repaired by 8-oxoguanine DNA glycosylase1 (OGG1) during base excision repair (BER) pathway. Because cellular response to oxidative challenge is accompanied by DNA damage repair, we tested whether the repair by OGG1 is compatible with transcription factor binding and gene expression. We performed electrophoretic mobility shift assay (EMSA) using wild-type sequence deriving from *Cxcl2* gene promoter and the same sequence bearing a single synthetic 8-oxoG at defined 5′ or 3′ guanine in runs of guanines to mimic oxidative effects. We showed that DNA occupancy of NF-κB present in nuclear extracts from tumour necrosis factor alpha (TNFα) exposed cells is OGG1 and 8-oxoG position dependent, importantly, OGG1 counteracting 8-oxoG outside consensus motif had a profound influence on purified NF-κB binding to DNA. Furthermore, OGG1 is essential for NF-κB dependent gene expression, prior to 8-oxoG excised from DNA. These observations imply that pre-excision step(s) during OGG1 initiated BER evoked by ROS facilitates NF-κB DNA occupancy and gene expression.

Arising through a plethora of biological matrices and a variety of settings, reactive oxygen species (ROS) are well recognized for representing a double-edged sword as both deleterious and beneficial species^1^. ROS cause predominantly base damages that do not occur randomly, and exhibit a strong bias distribution in genome^2^,^3^. Due to their lowest ionization potential among DNA components^4^, guanine bases, especially two- and more-base runs of guanines, e.g., 5′G < 5′GG < 5′GGG, are preferentially targeted by oxidants, and readily oxidizes to 7, 8-dihydro-8-oxo-2′-deoxyguanosine (8-oxoG)^5^,^6^. 8-OxoG is often used as a cellular biomarker to indicate the extent of oxidative stress^7^,^8^. Differing from normal guanine only by a single atom change, 8-oxoG does neither distort DNA helix structure nor perturb DNA or RNA polymerases function^9^. Such damage usually recognized precisely by 8-oxoguanine DNA glycosylase1 (OGG1) and repaired through base excision repair (BER) pathway^10^,^11^. Several studies have documented that the key searching step for damaged site in BER involves nonspecific DNA binding with induced destabilization by bending, and subsequent flipping of the lesion into the enzyme active site pockets^12^,^13^,^14^. OGG1 is a bifunctional DNA glycosylase endowed with an AP lyase activity. Following oxidized base removed, OGG1 cleaves DNA phosphodiester backbone at the 3′ side of the abasic site, producing a nick in the DNA backbone^15^,^16^.

Both DNA glycosylases and BER pathway are generally well conserved in evolution, tailoring to deal with the special features of base lesions. Most notably, the principal substrate for OGG1 recognizing is 8-oxoG paired with C, while a diverse array of oxidized DNA bases are repaired in the enzymic system, for example, hydantoin lesions are the best substrates for *Nei*-like DNA glycosylases (NEIL1–2)^17^,^18^,^19^. Additional studies reported that *Neil1*^−/−^ mice showed enhanced inflammatory responses upon chronic UV exposure relative to wild-type (Wt) controls^20^. By contrast, *Ogg1*^−/−^ mice exhibited lower expression of pro-inflammatory cytokines and inflammatory cells infiltration, even though resulting high levels of genomic 8-oxoG, embryonic development or life span were not affected^21^,^22^,^23^,^24^. The non-random distribution of 8-oxoG in genome and substrate specificity of OGG1 suggest that 8-oxoG and OGG1 initiated BER may play an important role in physiological and pathophysiological processes.

In addition to the extensive studies devoted to the role of oxidative nuclear DNA damage in coding sequences, the outcome of oxidative base lesions in non-coding regions remains to be elucidated. Intriguingly, vertebrate genome evolutionarily has a high GC content in the promoter regions of RNA polymerase II (RNA Pol II)-transcribed genes. Moreover, several transcription factors (TFs) (NF-κB, SP1, AP1 and NRF1) binding sites are rich in guanines, for example, the κB site recognized by NF-κB with a conserved sequence of 5′-GGGRNYYYCC-3′ (R is an unspecified purine; Y is an unspecified pyrimidine; and N is any nucleotide^25^), which are putatively vulnerable to oxidants. It is well documented that changes in *cis* elements result from oxidative modification may modulate the pattern of TFs binding^26^,^27^. In contrast to that *cis* effects, however, how DNA repair protein acting on oxidative bases affects promoter function remains elusive.

Here, we propose OGG1 counteracting 8-oxoG produced on key nucleotide in *Cxcl2* promoter benefits NF-κB binding to its consensus motif. Previously, we have shown that OGG1 and NF-κB/RelA coimmunoprecipitated from *Cxcl2* promoter^28^. But due to the major impediment to the ChIP-based evaluation is identifying genomic regions that interact with TFs but not individual binding sites^29^, it prompt us to address the issue in site resolution. In the present study, we generated several duplex oligonucleotides corresponding to *Cxcl2* gene promoter containing putative κB motif, one without (Wt) and the rest with single 8-oxoG situated at defined 5′ or 3′ guanine in runs of guanines to mimic ROS-induced oxidative damage occurrence. We performed electrophoretic mobility shift assay (EMSA) using nuclear extracts from mock and TNFα treated MLE-12 cells and recombinant proteins. We found that OGG1 recognizing 8-oxoG introduced outside cognate site facilitates NF-κB-DNA association robustly. The biological consequences of oxidants attack within *Cxcl2* promoter and the contribution of OGG1 counteracting 8-oxoG are facilitating NF-κB transcriptional activation. The results presented here provide novel insight into the ways in which organisms utilize oxidized guanines to regulate gene expression in a time-frame of DNA damage response.

## Results

### OGG1 plays a key role in providing the transcriptional activity from the *Cxcl2* promoter

**Figure 1:**
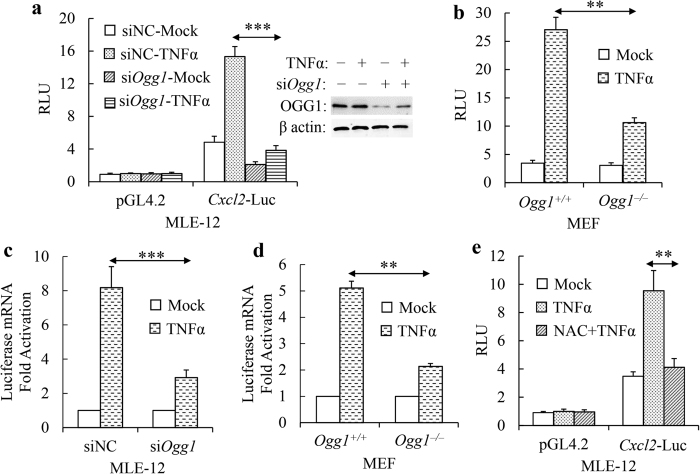
*Ogg1* depletion decreases *Cxcl2* promoter driven luciferase activity. (**a,b**) Luciferase expression driven by *Cxcl2* promoter is dependent on Ogg1 in MLE-12 (**a**), and MEF cells (**b**). All cells were exposed to TNFα for six hours. Insert: *Ogg1-*siRNA mediated Ogg1 knockdown in MLE-12 cells (WB analysis). (**c,d**) Decreased luciferase mRNA levels in *Ogg1*-siRNA transfected MLE-12 cells (**c**) and *Ogg1*^−/−^ MEF cells (**d**) upon TNFα exposure. Whole RNAs were isolated at 1 h post-exposure and luciferase mRNA levels were determined by RT-qPCR. (**e**) TNFα-induced promoter activation is inhibited by antioxidant pre-treatment in MLE-12 cells. RLU, relative luciferase unit. **p < 0.01, ***p < 0.001 (n = 3).

Our previous work has established that OGG1 can augment a subset of cytokine and chemokine gene expression in mouse lung^28^,^30^,^31^, including CXCLs, CCLs, ILs and TNFα. To confirm that OGG1 facilitates gene expression through promoter activation, we carried out small interfering RNA (siRNA)-mediated *Ogg1* knockdown and monitored how it affects luciferase activity. We were able to attain ~70% siOgg1 knockdown compared with control non-specific siRNA (Fig. 1a, insert). The activation of *Cxcl2* promoter revealed ~4 fold increase after 6 h of TNFα treatment, while in OGG1 deficient cells, inducible luciferase activity was significantly decreased (Fig. 1a). To confirm these results, we used *Ogg1*^−/−^ and *Ogg1*^+/+^ MEFs and showed that TNFα induced significantly less luciferase expression in *Ogg1*^−/−^ than wild type ones (Fig. 1b). To address the impact of OGG1 at transcription level, luciferase mRNA level was determined from reporter plasmids transfected cells over one hour TNFα exposure. RT-qPCR analyses showed a considerable increase of luciferase transcript in OGG1 expressing cells (~8 fold in MLE-12 cells, Fig. 1c; ~5 fold in MEF cells, Fig. 1d), while OGG1 depletion dampened transcript levels. Pre-treatment of cells with ROS scavenger N-acetyl-_L_-cysteine (NAC, GSH precursor) abrogated luciferase expression, suggesting oxidative DNA bases (e.g., 8-oxoG) were important for efficient *Cxcl2* gene expression in OGG1 expressing cells (Fig. 1e).

### OGG1 is essential for NF-κB-mediated transcriptional regulation

**Figure 2:**
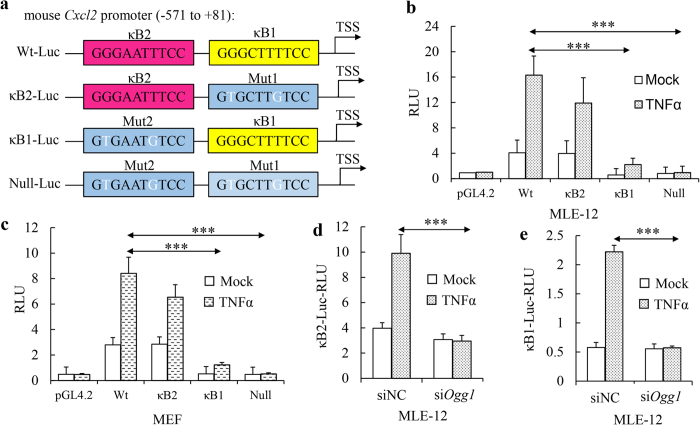
OGG1 is essential for NF-κB directed promoter activation. (**a**) Graphical depiction of *Cxcl2* promoter constructs: a wild-type and three mutated *Cxcl2* promoter fragments were cloned to drive luciferase expression as described in Materials and Methods. (**b,c**) Luciferase expression driven by Wt and mutated *Cxcl2* promoter. Constructs were transfected into MLE-12 (**b**) and MEF (**c**) cells. TNFα treated for 6 h and dual luciferase assays were performed as described in Materials and Methods. (**d,e**) Effect of Ogg1 on mutated Luc expression. κB2-Luc and κB1-Luc constructs were transfected into *Ogg1* expression and siRNA-mediated knockdown MLE-12 cells. TNFα treated for 6 h and dual luciferase assays were performed as described above. RLU, relative luciferase unit. ***p < 0.001 (n = 3).

Based on previous work, there are two NF-κB (−66 to −57, GGGAATTTCC, designated as κB2; −47 to −38, GGGCTTTTCC, designated as κB1) and one SP1 (−109 to −100, GGGGCGGGGC) putative binding sites immediately adjacent to TATA box in *Cxcl2* promoter^28^. To understand the functional role of potential *cis* regulatory elements in promoter activation, luciferase reporter gene constructs containing *Cxcl2* promoter fragments (depicted in Fig. 2a) and with one κB site mutation at each time were transfected into MLE-12 cells following with or without TNFa treatment. MLE-12 cells carrying the intact *Cxcl2* gene promoter (Wt-Luc) conferred strong TNFα responsiveness (Fig. 2b). The mutation of proximal κB site to a null sequence hardly affected inducible luciferase activity (Fig. 2b, κB2-Luc), while mutation of distal κB site abrogated inducible luciferase activity significantly (Fig. 2b, κB1-Luc), implying that κB2 site is indispensable for *Cxcl2* promoter activation. When both two κB sites were mutated to null sites, no inducible luciferase activity showed following TNFα treatment (Fig. 2b, Null-Luc), suggesting that NF-κB binding sites are crucial for *Cxcl2* promoter-driven gene expression. These reporter plasmid variants were also transfected to MEF cells, and the patterns were similar to those in MLE-12 cells (Fig. 2c).

To examine whether OGG1 acts on these κB sites differently, we carried out siRNA-mediated Ogg1 knockdown and monitored how it affects luciferase activity from mutated κB site reporter plasmids expressing cells. It showed that lacking of OGG1 decreased inducible luciferase activity both in κB2-Luc and κB1-Luc transfected cells (Fig. 2d,e). Of note, compared with non-treated situation, inducible luciferase activity in κB1-Luc transfected cells increased ~4-fold, implying other transcriptional factors, like SP1 participates in gene expression; OGG1 knockdown represses this induction to basal level, indicating that OGG1 may also regulate the performance of other *trans* acting factors.

### NF-κB-DNA associations are increased in nuclear extracts from OGG1 expressing cells

**Figure 3:**
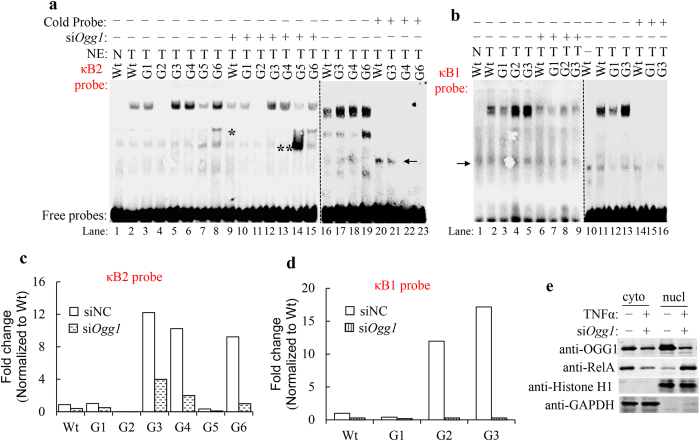
Effect of oxidative DNA damage on NF-κB binding present in nuclear extracts. (**a,b**) *Ogg1* depletion decreases levels of NF-κB-DNA association. MLE-12 cells were transfected either with negative control (NC) or *Ogg1* siRNA treated without (N) or with (T) TNFα for 30 min, and NE were prepared. NE (1 μg) were incubated with annealed oligonucleotides at RT for 5 min, and EMSA was carried out using κB2 oligoes (**a**) and κB1 oligoes (**b**). In some cases, binding was analysed with 100-fold unlabelled wild-type canonical κB oligonucleotide competitors. Arrowhead denote non-specific bands, * and ** indicates unknown bands. (**c,d**) Histogram of gel data shown in **a** (lanes 2–15) and **b** (lanes 2–9). The binding site occupancy of NF-κB on individual probes were calculated as a fold change in bands intensity compared to that of Wt-oligo using Image J software. (**e**) Independent nuclear translocation of p65. MLE-12 cells were transfected either with control or *Ogg1* siRNA. After 48 hour transfection, cells were exposed to TNFα for 30 min and fractionated into cytosolic and nuclear extracts. Western blot analysis with the indicated antibodies, Histone H1 was a nuclear fraction control and GAPDH was a cytosolic fraction control.

To examine whether NF-κB binds to the putative sites from *Cxcl2* promoter, we carried out electrophoretic mobility shift assay (EMSA) using seven double-stranded 33 bp oligonucleotides containing κB2 site (5′-GGAATTTCC-3′) and four double-stranded 31 bp oligonucleotides containing κB1 site (5′-GGCTTTTCC-3′). An efficient inducible activity was observed as strong retarded complex was detected using nuclear extracts (NE) from TNFα-exposed cells with κB2-Wt oligo, while NF-κB binding to same oligo was under detectable level in NE prepared from mock-treated cells (Fig. 3a, lanes 1 and 2). Compared with NF-κB-Wt oligo complex, 8-oxoG modified at G1 showed nearly equal binding, while κB2-G2 showed no retarded complex, implying that OGG1 recognizing 8-oxoG within κB site competes with NF-κB on motif binding (Fig. 3a, lanes 3 and 4). The level of protein-DNA complex formed with 8-oxoG modified upstream of κB site was far greater than Wt, ~12-fold using κB2-G3 and G4; G6 8-oxoG modified at reverse strand bind NF-κB ~10-fold to Wt (Fig. 3a, lanes 5, 6 and 8). On the contrary, κB2-G5 8-oxoG, just adjacent κB site modified at reverse strand, showed slightly decreased NF-κB binding over that of Wt (Fig. 3a, lane 7). In order to test if OGG1 in NE was responsible for altered NF-κB binding, we carried out siRNA-mediated *Ogg1* knockdown and tested the effects on NF-κB-DNA association. We found that the inducible NF-κB-DNA association significantly decreased (κB2-G3, G4 and G6 oligoes) to those of OGG1 expressing ones (Fig. 3a, lanes 12, 13 and 15). Whether OGG1 depleted or not, NF-κB binding to κB2-G2 is not affected (Fig. 3a, lane 11). These extensive retarded complexes were specific NF-κB contained, as in each case, binding was blocked by unlabelled probe competitor (Fig. 3a, lanes 20–23).

In the case of κB1 oligoes, Wt oligo shifted obvious retarded complex with TNFα-treated NE; 8-oxoG introduced at G1 decreased that association slightly, while G2 and G3 8-oxoG introduced outside κB site shifted ~17-fold NF-κB to Wt (Fig. 3b, lanes 1–9). Competition assay showed those protein-DNA complexes were competed out by cold probe (Fig. 3b, lanes 14–16).

In case that deficient NF-κB binding was due to the impairment of p65 translocation, we tested p65 protein level in the cytoplasmic and nuclear fraction from Ogg1 knockdown cells. Westernblotting revealed that cytoplasmic levels of p65 decreased and nuclear levels increased after induction, suggesting that OGG1 does not govern p65 nuclear importation (Fig. 3e). Taken together, these observations suggest a dominant role of OGG1 in modulating NF-κB binding to its DNA targets.

### OGG1-DNA interactions enhance NF-κB binding synergistically

**Figure 4:**
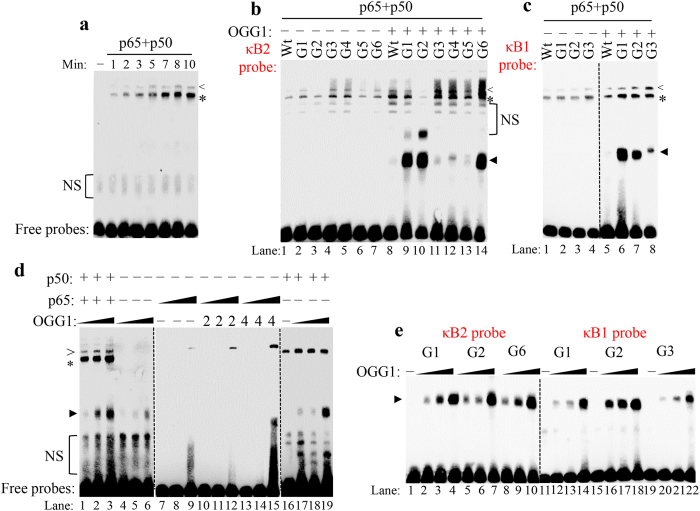
OGG1 enhances NF-κB-DNA association. (**a**) Kinetic NF-κB homo- and heterodimer binding to κB2-Wt oligo. EMSA shows purified p65 (3.75 ng/sample) and p50 (2.75 ng/sample) proteins binding to κB2-Wt probe over a 10 min time course. (**b,c**) Effects of OGG1 encountering 8-oxoG on NF-κB home- and heterodimer binding to κB2-oligoes (**b**) and κB1-oligoes (**c**). Purified p65 (3.75 ng/sample) and p50 (2.75 ng/sample) were pre-incubated and oligoes were added with or without purified OGG1 (4 ng/sample) after 10 min incubation. (**d**) The effect of OGG1 on NF-κB subunit binding to κB2-G6 oligo. Lanes 1–3, recombinant p65 (3.75 ng/sample) and p50 (2.75 ng/sample) were incubated with increasing amounts of OGG1 (1, 2 and 4 ng), and then mixed with oligo at RT for 10 min. Lanes 4–6, increasing amounts of OGG1 (1, 2 and 4 ng) binding to κB2-G6 oligo. Lanes 7–15, pre-incubated p65 (3.75, 7.5 and 15 ng respectively) were mixed alone or with 2 or 4 ng of purified OGG1, followed by adding κB2-G6 oligo. Lanes 16–19, pre-incubated p50 (2.75 ng/sample) were mixed with increasing amounts of OGG1 (1, 2 and 4 ng respectively), followed by an EMSA with κB2-G6 oligo. <denotes p65/p50 heterodimer-DNA complex, *denotes p50/p50 homodimer-DNA complex, triangle indicates OGG1-DNA complex, NS denotes non-specific bands. (**e**) Purified OGG1 binds to 8-oxoG containing DNA. EMSA was performed using increasing concentrations of purified OGG1 (0, 2, 4 and 8 ng respectively) incubated with indicated 8-oxoG containing oligoes at RT for 10 min.

To provide additional evidence for OGG1 modulating NF-κB binding through interaction with DNA, we utilized recombinant NF-κB subunits and performed EMSA either singly or in the presence of purified OGG1. Purified p65 and p50 proteins were pre-incubated at 37 °C for 60 min, so that p65/p50 heterodimer and p50/p50 homodimer were allowed to form^32^, after that EMSA was carried out with κB2-Wt oligo added at RT within 10 min time course. Results showed that p50/p50 homodimer binds the probe from 1 min on, and gradually increase until 10 min, while obvious p65/p50 heterodimer-DNA complex appeared at 5 min and then approached an equilibrium until 10 min (Fig. 4a). Similar data were obtained using κB1-Wt oligo (data not shown). Therefore, we chose 10 min as the incubation time for analysing the binding properties of purified protein in EMSA.

Figure 4b shows that p50/p50 homodimer bound to κB2 oligoes with varying efficiency, and p65/p50 heterodimer recognizes oligoes with bias, preferring κB2-G3 and G4 rather than κB2-G2 (Fig. 4b, lanes 1–7). After adding purified OGG1, the association of both homo-and heterodimer with oligoes (except κB2-G2) was stronger than NF-κB alone (Fig. 4b, lanes 8–14). Especially, NF-κB binding to κB2-G3, G4 and G6 exhibited higher level than Wt, implying OGG1 encountering 8-oxoG outside κB motif increases NF-κB binding to DNA. In the presence of OGG1, NF-κB binding to κB2-G2 was decreased compared to Wt, suggesting 8-oxoG introduced within κB motif and recognized by OGG1 would compromise NF-κB binding. NF-κB alone could recognize four κB1 oligoes (Fig. 4c, lanes 1–4), after adding OGG1, both homo- and heterodimer binding were increased, among which κB1-G3 showed highest shifted amount (Fig. 4c, lanes 5–8).

As we showed above, OGG1 significantly affects NF-κB binding to κB2-G6 oligo and efficiently bound to that oligo itself, we chose κB2-G6 for further studies. Purified p65 and p50 were pre-incubated, followed by mixing with increasing amounts of OGG1 and EMSA was performed. It showed that the occupancies of both hetero- and homodimer on κB2-G6 oligo were increased in OGG1concentration dependent manner (Fig. 4d, lanes 1–3). Amazingly, OGG1-DNA complex was significantly less in absence of NF-κB (Fig. 4d, lanes 4–6). We can conclude from these results that there is a bidirectional interaction between OGG1 and NF-κB, OGG1 facilitates NF-κB assembly on DNA and NF-κB reciprocally increases OGG1-DNA association. To examine the effect of OGG1 on NF-κB subunit, p50 and p65 were pre-incubated individually and then mixed with purified OGG1. p65 alone bound to oligo only at high concentration (15 ng/sample) (Fig. 4d, lane 9), which may represent p65/p65 homodimer^33^. Importantly, DNA occupancy of recombinant p65 increased more than 10-fold in the presence of OGG1 (Fig. 4d, lanes 12 and 15). Interestingly, OGG1 didn’t shift κB2-G6 oligo with low concentration of p65 (Fig. 4d, lanes 11 and 12, 13 and 14), only at 15 ng of purified p65 could show obvious OGG1-DNA complex (Fig. 4d, lanes 12 and 15). The level of p50/p50 homodimer-DNA complex was enhanced mildly by the lowest OGG1 concentration (1 ng/sample), which was not further increased by higher amounts (Fig. 4d, lanes 16–19). OGG1-mediated oligo shifts were observed at indicating concentration. Surprisingly, purified OGG1 didn’t affect NF-κB-DNA complex migration, instead, independent OGG1 shifts were observed with 8-oxoG containing oligoes (Fig. 4b, c and d).

In independent experiments, the ability of purified OGG1 binding to 8-oxoG-introduced κB2 and κB1 oligoes were also determined (Fig. 4e). Owing to insufficient flanking bases for OGG1 docking in κB2-G3, G4 and G5 oligoes, OGG1 shifts those oligoes with higher amounts (data not shown).

### Transient accumulation of genomic 8-oxoG is crucial for efficient expression of NF-κB target genes

**Figure 5:**
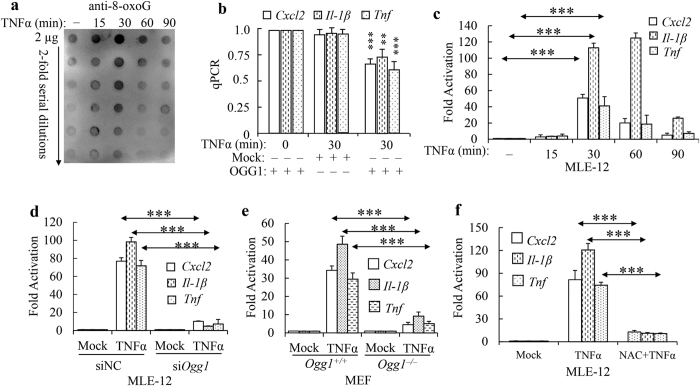
OGG1 is required for a subset of pro-inflammatory gene expression upon TNFα stimulation. (**a**) Changes in global 8-oxoG levels assayed by dot blot analysis. Genomic DNA was isolated at times indicated and immunoblotted as described in Materials and Methods. (**b**) Changes in OGG1 substrate levels in *Cxcl2, Tnf and Il-1β* proximal promoter regions after TNFα challenge. DNAs were isolated at 0 and 30 min after TNFα exposure and promoter regions were amplified by RT-qPCR after mock (−) and OGG1 digestion (+). (**c**) Time course of gene expression upon TNFα exposure of MLE-12 cells. RNAs were isolated at times indicated and mRNA levels were determined by RT-qPCR. (**d,e**) *Ogg1* depletion decreases gene expression in MLE12 (**d**) and MEF (**e**) cells. Parallel cultures of MLE12 cells were transfected with control or *Ogg1*-siRNA, after which TNFα treated for 30 min. RNAs were isolated at times indicated and mRNA levels were determined by RT-qPCR. (**f**) TNFα-induced gene activation is inhibited by antioxidant pre-treatment. ***p < 0.001 (n = 3).

We next examined if genomic 8-oxoG production is compatible with gene expression. Examination of endogenous 8-oxoG gave a congruent result^34^,^35^: dot blot analysis showing that TNFα exposure of MLE-12 cells leads to global increase in 8-oxoG level from 15 min on, with a peak at 30 min and lost progressively at 90 min (Fig. 5a). To ascertain 8-oxoG and related base oxidation products accumulated in promoter regions of *Cxcl2, Tnf* and *Il-1β*, “Flare”-qPCR was applied^36^. DNA was isolated at 0 or 30 min after TNFα exposure, and mock- or OGG1-digested prior to PCR amplification. Compared to mock, OGG1 digestion substantially decreased the PCR products in DNA extracted from TNFα exposed cells (Fig. 5b). We tested whether the kinetics of 8-oxoG production correlates with gene expression through three representative NF-κB dependent genes. Results showed that *Cxcl2, Tnf* and *Il-1β* mRNA levels were increased from 15 min on and peaked at 30 min after TNFα exposure (Fig. 5c). To confirm that OGG1 is required for TNFα induced rapid gene expression, we carried out siRNA mediated Ogg1 knockdown in MLE-12 cells and compared mRNA level to control siRNA transfected ones. RT-qPCR showed that OGG1 depletion in TNFα-stimulated MLE-12 cells abrogated the induction of indicated gene mRNA (Fig. 5d). Similar results obtained upon TNFα challenged Ogg1 deficient MEF cells (Fig. 5e). To clarify the predominant role of OGG1 in our experimental model, uracil N-glycosylase (UNG) (removes uracil by deamination of cytosine in DNA)^37^ was down-regulated by siRNA. *Ung* deficiency did not impair prompt up-regulation of target genes in MLE-12 cells (data not shown). NAC pre-treatment of cells decreased mRNA expression to nearly basal level, indicating NF-κB activation is dampened by antioxidant (Fig. 5f). These data suggest that both OGG1 and its substrate are required for transcriptional events.

## Discussion

Many reported that ROS are the central effectors in a variety of physiological processes^38^,^39^,^40^,^41^. Much attention have been caught by the role of ROS in posttranslational modifications of TFs (e.g., NF-κB, AP1)^34^,^42^,^43^, however, one background is neglected during transcriptional regulation, that is the simultaneous oxidative damage to cellular macromolecules, especially DNA base lesions that occur through oxidative reactions. Taking that not only activation of transcription factors and their nuclear translocation, but their access to the binding motifs in the chromatin is actively controlled, 8-oxoG and its repair complex (OGG1-DNA interactosome) perform in gene regulatory regions could provide another insight in gene expression.

What we demonstrate here is simulating one way of mediating transcription through OGG1-DNA interactosome. Implicit in that notion are extensions of three: first, 8-oxoG in response elements themselves can act as *cis* effects that has either no effect, induces a full or partial inhibition or enhances TFs binding; second, OGG1 surveying the genome for damaged bases can act as *trans* effects that facilitates TFs binding to DNA synergistically; third, the combination of 8-oxoG and OGG1 determines the levels of TFs binding, in part by which selective gene transcription may be achieved.

We observed varying NF-κB binding as a function of 8-oxoG in oligoes, which is not surprising as the *cis* effects are the most studied and offer a good argument for the significant role of oxidative damage interference in promoter functions. 8-OxoG formation in DNA response elements has the capacity to interfere with normal gene regulation on the basis of changes in the binding affinity of TFs. The affinity changes are either increased, unchanged, or diminished that depend on various factors, including the particular TF-promoter system under investigation (e.g., SP1, AP1, or NF-κB) and the precise site of damage within the consensus binding motif^26^,^27^. Although the mechanism(s) of affinity changes are not well defined, they most likely involve 8-oxoG:C pairs showing more increased local and global flexibility than G:C^44^.

Another more intriguing aspect of DNA damage outcome on gene regulation involves *trans* effects from DNA binding proteins. In exposure of oxidative stress, damage-specific DNA glycosylase OGG1 searches oxidized guanines without any delay and initiates BER, through which small base lesions that do not significantly distort DNA double helix are corrected. Previously, we have shown that OGG1 is non-productively bound to oxidative lesions and its excision activity is inhibited in response to TNFα treatment^28^. In the present study, we showed that OGG1 could facilitate NF-κB binding to its DNA targets through recognizing 8-oxoG outside consensus motif, the mechanism by which is unknown at present. The crystallographic observation of OGG1-DNA complex shed some light upon the biological effects of OGG1 on modifying the allosteric effect of DNA. Verdine and co-workers have revealed that OGG1 bends DNA sharply when it is bound to an 8-oxoG containing site, giving rise to a pronounced kink in duplex, and more importantly, also showed that OGG1 induces similar bending when it binds to undamaged sites (~70°)^45^,^46^,^47^. Impressively, a universal feature of most DNA repair glycosylases, especially for OGG1, is the free energy cost for DNA bending during base flipping since it takes place in the presence of only protein, DNA, and the cofactor S-adenosylmethionine^13^,^48^. It is quite remarkable that bending or kinking of DNA facilitates NF-κB binding, presumably because flexible duplex structure overcomes the energy barrier upon bending^49^. We suspect that OGG1 bent DNA when searching for damaged sites, lowering the energy cost for NF-κB twisting DNA and creating a stereo-specific interface that suited NF-κB recognizing consensus motif. Deficiency in UNG did not decrease pro-inflammatory gene expression upon TNFα exposure encouraged us to test uracil levels deaminated from cytosine in the future. Our results showing OGG1 counteracting 8-oxoG situated outside κB motif increased NF-κB-DNA association indicate that much larger segments than consensus recognition sites need to take into account for individual TF function. Note that DNA glycosylases process on genome without sequence bias^13^,^45^, we observed OGG1 regulates promoter activity at both canonical and non-canonical κB sites.

The combination of 8-oxoG and OGG1 determines the levels of TFs binding on DNA. 8-OxoG limits the position of OGG1 where TFs binding would be benefited. In that case, OGG1 recognizing 8-oxoG produced within TFs binding site shoves corresponding TF by competition, while outside motifs facilitates TFs binding robustly. The final outcome for TF binding in response to stimuli might compromise those two parts *in vivo*.

It’s still an enigma that purified OGG1 didn’t slower the mobility of NF-κB-DNA complex in EMSA. One possibility is that the high mobility of OGG1 ensures its availability throughout the genome and makes the transcription apparatus highly dynamic, which is the common property for nuclear proteins^50^. TFs binding to consensus motifs normally proscribed by the energetic cost of DNA bending and twisting within its persistence length, the function of OGG1 is more like an “architectural” protein that allows TF-DNA interactions. Carey also suggested that architectural proteins can be bypassed if the strength or flexibility of the interactions can absorb the energy cost of DNA distortion^51^.

In conclusion, we were alert for the possibility that DNA glycosylases are the first sensor detecting physiological changes and taking rapid responses, beckoning TFs like NF-κB to locate their sites. Our data provided here may be viewed that oxidation of guanines in gene regulatory regions may be utilized by transcriptional machinery. Our future goal is to ascertain the exact DNA conformational changes caused by specific glycosylase that are required for TF binding, and the potential cooperative action(s) of other substrate-specific DNA glycosylases function in gene regulation. Owing to the paucity of our knowledge concerning the function of OGG1 in transcriptional response and the likelihood that increased knowledge of TFs will lead to increased insight into the causes of human diseases, it is of utmost importance that we expand our understanding of how site-specific DNA glycosylases contribute to gene regulation.

## Methods and Materials

### Cell culture and treatments

MLE-12 (an immortalized type 2 mouse lung epithelial cell line), Ogg1^−/−^ and Ogg1^+/+^ mouse embryo fibroblasts (MEFs) were kindly provided by Professor Istvan Boldogh (University of Texas Medical Branch at Galveston, Texas). Cells at about 80% of confluence were treated with 20 ng/ml recombinant human TNFα (Cat # H8916; Sigma) for indicated time course at 37 °C. In the case of antioxidant treatment, cells were pre incubated for 1 h in the presence of 10 mM N-acetyl cysteine (NAC) (Cat # A7250; Sigma)^52^ followed by TNFα treatment.

### Depletion of target gene expression

For RNA interference experiments, scramble control or smart-pool siRNA against *Ogg1* and *Ung* (Dharmacon) were transfected into MLE-12 using Lipofectamine 2000 (Invitrogen) as previously described^28^. Cells were used for experiments after 48 h of incubation, and target gene knockdown efficiency was validated by Western blot.

### Luciferase reporter assays

Wt-Luc containing mouse *Cxcl2* promoter (−571 to +81) was generate as previously described^28^ and mutant versions κB2-Luc and κB1-Luc were created using Fast Mutagenesis System (Cat # FM111, TransGen Biotech). MLE-12, wild-type and Ogg1 deficient MEF were transiently transfected with luciferase reporter DNA and Renilla luciferase expression plasmid was co-transfected as an internal control. The total amount of plasmid DNA was kept constant for all assays. Transient transfections were carried out using Lipofectamine 2000 (Invitrogen) and luciferase activities of total cell lysates were measured using the dual-luciferase reporter assay system (Promega) following the manufacturer’s instructions.

### Preparation of cell nuclear extracts (NE)

The preparation an extract from nuclei was made using CelLytic^TM^ NuCLEAR^TM^ Extraction Kit (Cat # NXTRACT, Sigma) and total protein was quantified by Pierce BCA Protein Assay Kit (Cat # 23225, Thermo Scientific).

### Electrophoretic mobility shift assay (EMSA)

Consensus oligonucleotides containing the binding sites for NF-κB were synthesized at Sangon Biotech (Shanghai, China), and site-specific guanine residue within each of the oligonucleotide was replaced with single 8-oxoG modification (Table 1). EMSA was performed using LightShift^®^ Chemiluminescent EMSA Kit (Cat # 20148, Thermo Scientific). For binding reactions, 10 fmol Biotin-labeled probe was mixed with 1 μg NE in a total volume of 10 μl containing 10 mM Tris-HCl (pH 8.0), 5 mM NaCl, 1 mM EDTA, 1 mM DTT, 0.1 mg/ml BSA and 0.1 μg/μl Poly[d(I-C)]^53^. p50/p65 hetero-complexes were made by adding 2.75 ng of p50 (Cat # AG-40T-0021, Adipogen Corp) and 3.75 ng of p65 (Cat # AG-40T-0020, Adipogen Corp) purified protein, followed by incubation at 37 °C for 60 min^32^. After that, the mixture was incubated with probe with or without indicated purified OGG1 (Cat # ENZ-253, ProSpec). Binding was performed for 5–10 min at room temperature (RT). The reaction mixtures were applied to a 6% polyacrylamide gels in low-ionic-strength buffer (0.5xTBE) and electrophoresed for 2 h at 4 °C.

### Dot blot analysis

Genomic DNA was isolated using a Qiagen Gentra Puregene Kit or the phenol-chloroform-isoamyl alcohol method. RNase A digestion was included in the isolation procedure. Isolated genomic DNA (2 μg per sample) was denatured in 0.1 M NaOH for 5 min at 95 °C, and chilled rapidly on ice. Samples were serially diluted twofold and spotted on a positively charged nitrocellulose membrane using a Bio-Dot Microfiltration Apparatus (Cat # 170-6545, Bio-Rad). The blotted membrane was washed in 2 × SSC buffer, and UV cross-linked. The membrane was then blocked in Odyssey buffer (Li-Cor) diluted 1:1 in PBS for 1 hour at RT. Mouse anti-8-oxoG monoclonal antibody (Cat # MOG100 P, Japan Institute for the Control of Aging) in Odyssey:PBS was added for 1 hour at RT. The membrane was washed for 10 min three times in PBS, and then incubated with HRP-conjugated sheep anti-mouse immunoglobulin-G (IgG) (GE, 1:5,000) secondary antibodies in Odyssey:PBS for 1 h at room temperature. The membrane was then washed for 10 min three times in PBS and visualized by chemiluminescence with GE ECL Plus.

### Flare qPCR

Oxidative modified base products in promoter regions were determined by OGG1 digestion-coupled qPCR (Flare-qPCR)^28^. Briefly, cells were exposed to TNFα for 30 min or not, DNA was isolated and 2 μg was mock- or OGG1 (5 ng) digested. After phenol/chloroform extraction 20 ng DNA was subjected to qPCR. Primers: *Tnf* : F, 5′-AACTCTCAAGCTGCTCTGCC-3′ R, 5′-CAAGGAATCTCCTCCCCGTC-3′ (−499 to −138); *Cxcl2*: F: 5′- GCTCAGTACACCGCAGGAAC-3′, R: 5′-CTGCCCTTCCACTATGGGAC-3′(−531 to −186), *Il-1β*: F: 5′-AAGGAAGTGCGTGTCTCTCC-3′ R: 5′-TCAAGGGGTGGCAGATAGTG-3’ (−567 to −251).

### Quantitative RT-PCR (RT-qPCR)

Total RNA was isolated from cultured cells using Qiagen RNeasy with on-column DNase I treatment. Complementary DNA (cDNA) was generated from 1 μg of RNA using random hexamers to prime the reaction. The cDNA was used as template for RT-qPCR. RT-qPCR was performed in combination with the SYBR Green qPCR Master Mix (Cat# 638320, TaKaRa). Relative expression levels of target genes were calculated by ΔΔCt method. The relative amount of each gene was normalized using βactin and Tubulin housekeeping genes.

### Statistical tests

Results were tested for statistical significance using one-way ANOVA to analyse changes at mRNA levels. All data values are presented as mean ± SD. *P < 0.05, **P < 0.01, ***P < 0.001.

## Additional Information

**How to cite this article**: Pan, L. *et al*. OGG1-DNA interactions facilitate NF-κB binding to DNA targets. *Sci. Rep.* **7**, 43297; doi: 10.1038/srep43297 (2017).

**Publisher's note:** Springer Nature remains neutral with regard to jurisdictional claims in published maps and institutional affiliations.

## Figures and Tables

**Table 1:** The sequence of oligoes used in this study.

Oligo Sequences	Site
5′-TGAG^3^GGG^4^ACCCTGAGCTCA **G**^1^**GG**^2^**AATTTCC** CTGG-3′	κB2
3′-ACTCCCCTGGG^6^ACTCGAGT **CCCTTAAAGG** G^5^ACC-5′ Biotin	
5′-TTCCCTGGTCCCC **G**^1^**GGCTTTTCC** AGACATCG-3′	κB1
3′-AAGGG^3^ACCAGGGG^2^ **CCCGAAAAGG** TCTGTAGC-5′ Biotin	

Consensus motif is in bold. Wt refers to no 8-oxoG modified oligoes, and the position of 8-oxoG is shown by numbers above. Each oligo contains a single 8-oxoG modification. The κB2 oligoes contain classic κB motif GGGAATTTCC, named κB2-G1 to G6; κB1 oligoes contain GGGCTTTTCC binding site named κB1-G1 to G3, differing only by a cytosine in the position 4 from the canonical sequence GGGRNWYYCC (R = Purine, W = A or T, N = Any nucleotide, Y = Pyrimidine)^25^.
